# Boundary effects across filter spatial scales

**DOI:** 10.1186/1471-2202-15-S1-P19

**Published:** 2014-07-21

**Authors:** Calden Wloka, Neil Bruce, John Tsotsos

**Affiliations:** 1 Electrical Engineering and Computer Science, York University, Toronto, ON, Canada, M3J 1P3; 2 Centre for Vision Research, York University, Toronto, ON, Canada, M3J 1P3; 3 Department of Computer Science, University of Manitoba, Winnipeg, MB, Canada, R3T 2N2

## 

Most saliency algorithms rely on a filter processing stage in which an image is analyzed using a bank of convolution kernels. When applying a convolution to an image, however, a region of pixels with thickness equal to one-half the kernel width at the image border is left undefined due to insufficient input (this undefined region is hereafter referred to as the *boundary region*). While the percentage of the output image falling within the boundary region is often kept small, this limits the spatial scale of filter which can be applied to the image. There is clear psychophysical evidence from visual search tasks that spatial scale can be used as a component of visual search, with differences in feature size, spatial frequency, and sub-component grouping [1]. Thus, handling filters with dimensions that are significant with respect to the image size is worthwhile if the spatial scale component of visual search is to be effectively incorporated, but this requires dealing with the resulting boundary region.

A large number of computational strategies have been developed over the years for dealing with the boundary region issue, including: image tiling/wrapping, image mirroring, image padding, filter truncation, and output truncation. Formal evaluations and comparisons of such strategies have not previously been performed. We provide such a comparison using visual search stimuli commonly utilized in human psychophysical experiments, as well as propose a novel method for incorporating information across multiple spatial scales with an output image defined up to the boundary region created by the smallest spatial scale.
